# Particulate matter pollution and older adult health: global trends and disparities, 1991–2021

**DOI:** 10.3389/fpubh.2024.1478860

**Published:** 2024-11-06

**Authors:** Qiong Yi, Min Liu, Dandan Yan, Xu Wang, Deqian Meng, Ju Li, Kai Wang

**Affiliations:** ^1^Department of Rehabilitation, The Affiliated Huaian No.1 People's Hospital of Nanjing Medical University, Huaian, China; ^2^Department of Rheumatology and Immunology, The Affiliated Huaian No.1 People's Hospital of Nanjing Medical University, Huaian, China

**Keywords:** particulate matter pollution, older adult, ambient particulate matter pollution, household air pollution from solid fuels, mortality

## Abstract

**Background:**

Particulate matter pollution (PMP) is a major global health concern, with the older adult being particularly vulnerable. This study aimed to analyze global trends in PMP-related deaths and disability-adjusted life years (DALYs) among the older adult from 1991 to 2021.

**Methods:**

Using data from the Global Burden of Disease Study 2021, we examined the impacts of ambient particulate matter pollution (APMP) and household air pollution from solid fuels (HAP-SF). We analyzed trends across different regions, socioeconomic development levels, age groups, and genders.

**Results:**

APMP-related older adult deaths increased from 1,745,000 to 3,850,000, and DALYs from 32,000,000 to 70,000,000. However, age-standardized mortality rate decreased from 384 to 337 per 100,000. HAP-SF-related deaths decreased from 2,700,000 to 2,100,000, and DALYs from 54,000,000 to 42,000,000. Age-standardized mortality rate for HAP-SF declined from 580 to 188 per 100,000. High APMP burden was concentrated in Asia, Africa, and the Middle East, while high HAP-SF burden was found in parts of Africa and South Asia. East Asia had the highest APMP-related older adult deaths (1,680,000) with an age-standardized mortality rate (ASMR) of 619 per 100,000. For HAP-SF, South Asia bore the heaviest burden with 1,020,000 deaths and an ASMR of 616 per 100,000. Females consistently experienced higher age-standardized DALYs rate than males for both APMP and HAP-SF across all regions and years. APMP burden showed a weak negative correlation with the Socio-demographic Index (SDI) at the regional level (*r* = −0.25, *p* < 0.001) but no significant correlation at the country level. HAP-SF burden exhibited strong negative correlations with SDI at both regional (*r* = −0.74, *p* < 0.001) and country levels (*r* = −0.83, *p* < 0.001).

**Conclusion:**

Despite overall improvements, PMP continues to significantly impact older adult health globally, with substantial regional and gender disparities. These findings emphasize the need for targeted interventions, particularly in developing regions, and continued global efforts in air quality improvement and clean energy promotion.

## Introduction

Air pollution has emerged as a major global public health concern, with particulate matter pollution (PMP) having particularly significant impacts on human health (1). Particulate matter pollution (PMP) can be broadly categorized into outdoor (ambient) and indoor pollution. Ambient particulate matter pollution (APMP) primarily originates from outdoor sources such as vehicle emissions and industrial activities. Indoor PMP includes various sources within enclosed spaces, with household air pollution from solid fuels (HAP-SF) being a significant component, especially in developing countries (2, 3). While recent acceleration in globalization and industrialization has exacerbated APMP issues globally, HAP-SF continues to pose a serious health threat, especially in developing countries (4). These two forms of pollution present distinct challenges across different regions and socioeconomic contexts, necessitating targeted approaches in addressing their health impacts.

The older adult, being a vulnerable population, are highly susceptible to air pollution (5). Studies indicate that long-term exposure to particulate matter may accelerate lung function decline in older adults and increase morbidity and mortality rates for cardiovascular and respiratory diseases (6).

Research by Cohen et al. revealed that in 2015, approximately 4.2 million premature deaths globally were attributable to PM2.5 exposure, with a significant proportion among the older adult (7). As global population aging intensifies, the impact of particulate matter pollution on older adult health has garnered increasing attention (8). The World Health Organization (WHO) reflected the urgent need for air quality improvement in its 2021 updated guidelines, lowering the annual PM2.5 concentration limit from 10 μg/m^3^ to 5 μg/m^3^.

However, long-term trend studies on the health effects of particulate matter pollution on the older adult remain insufficient. Stanaway et al. analyzed changes in deaths and disease burden caused by air pollution from 1990 to 2017 using Global Burden of Disease Study (GBD) data, but did not specifically focus on the older adult population (9). Li et al.’s research, centered on older adult Chinese, found a significant positive correlation between PM2.5 concentration and older adult mortality rates from 2013 to 2017, but lacked a global perspective (10).

Given this context, our study aims to comprehensively analyze global trends in older adult deaths and disability-adjusted life years (DALYs) caused by particulate matter pollution (including APMP and HAP-SF) from 1991 to 2021. Utilizing the latest GBD 2021 research data, we will explore differences between regions, countries with varying levels of socioeconomic development, and the influence of age and gender factors.

The significance of this study is threefold: (1) It evaluates the effectiveness of global particulate matter pollution mitigation efforts over the past three decades through long-term trend analysis; (2) It provides a basis for formulating targeted air quality improvement strategies by comparing changes in APMP and HAP-SF impacts; and (3) By focusing on the older adult population, it offers important references for addressing environmental health challenges in the context of population aging. The results will aid policymakers, public health experts, and environmental scientists in better understanding and addressing the impact of particulate matter pollution on older adult health.

## Methods

### Data source and processing

This study leveraged data from the GBD 2021 study, which provides comprehensive information on deaths and DALYs across various demographic and geographic categories. Our analysis specifically targeted the older adult population, defined as individuals aged 60 years and above. The older adult cohort was further stratified into eight age groups: 60–64, 65–69, 70–74, 75–79, 80–84, 85–89, 90–94, and 95+ years.

### Statistical analysis

Age-standardized rates (ASRs) for incidence, prevalence, deaths, and DALYs were computed using the WHO’s 2000–2025 World Standard Population as the reference. The ASR calculation followed a four-step process: (1) collection of raw incidence numbers and population data for each region, year, and age group; (2) computation of age-specific incidence rates; (3) calculation of weighted incidence rates for each age group; and (4) summation of weighted incidence rates to obtain the total ASR. The ASR was expressed as: ASR = *Σ*(w[i] * r[i]) / Σw[i]. Where w[i] represents the weight for age group i in the standard population, and r[i] denotes the age-specific rate for age group i in the population of interest.

To quantify long-term trends in PMP-related older adult deaths and DALYs from 1990 to 2021, we employed the estimated annual percentage change (EAPC) metric. The EAPC is a widely used measure in epidemiological studies to describe trends over time, providing a single, easily interpretable number that represents the average rate of change per year. We calculated the EAPC through the following steps: (1) Data Transformation: We first transformed the ASR for each year using a natural logarithm; (2) Linear Regression: We then fitted a linear regression model to these transformed data points: ln(ASR) = *β*0 + β * (year − 1990) + *ε*. Where β0 is the intercept, β is the slope, and ε is the error term; (3) EAPC Calculation: Finally, we calculated the EAPC using the formula: EAPC = (e^*β* − 1) * 100%. This formula converts the slope (*β*) from the log scale back to a percentage, representing the average annual percent change; (4) To assess the precision of our EAPC estimates, we calculated 95% uncertainty intervals (UI): 95% UI = [(e^(*β* − 1.96 * SE) − 1) * 100%, (e^(β + 1.96 * SE) − 1) * 100%]. Where SE is the standard error of β. A positive EAPC value indicates an upward trend in ASR during the study period. A negative EAPC value suggests a downward trend. The magnitude of the EAPC represents the average annual percentage change.

All statistical analyses and visualizations were conducted using R software (version 4.3.3). Key R packages employed included “reshape2” for data reshaping, “tidyverse” for data manipulation and visualization, and “ggplot2” for graphic creation. Statistical significance was set at *p* < 0.05.

## Results

### Overall trends in particulate matter pollution’s impact on older adult health

**Figure 1:**
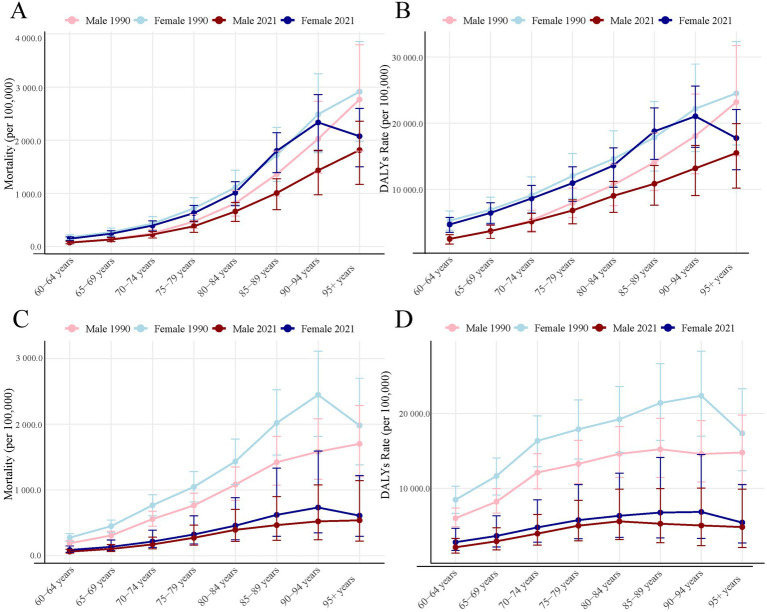
PMP-related older adult crude mortality and DALYs rate in 1990 and 2021 by sex and age group. **(A)** APMP-related older adult crude mortality; **(B)** APMP-related older adult crude DALYs rate; **(C)** HAP-SF-related older adult crude mortality; **(D)** HAP-SF-related older adult crude DALYs rate. PMP, particulate matter pollution; APMP, ambient particulate matter pollution; HAP-SF, household air pollution from solid fuels; DALYs, disability-adjusted life years.

**Figure 2:**
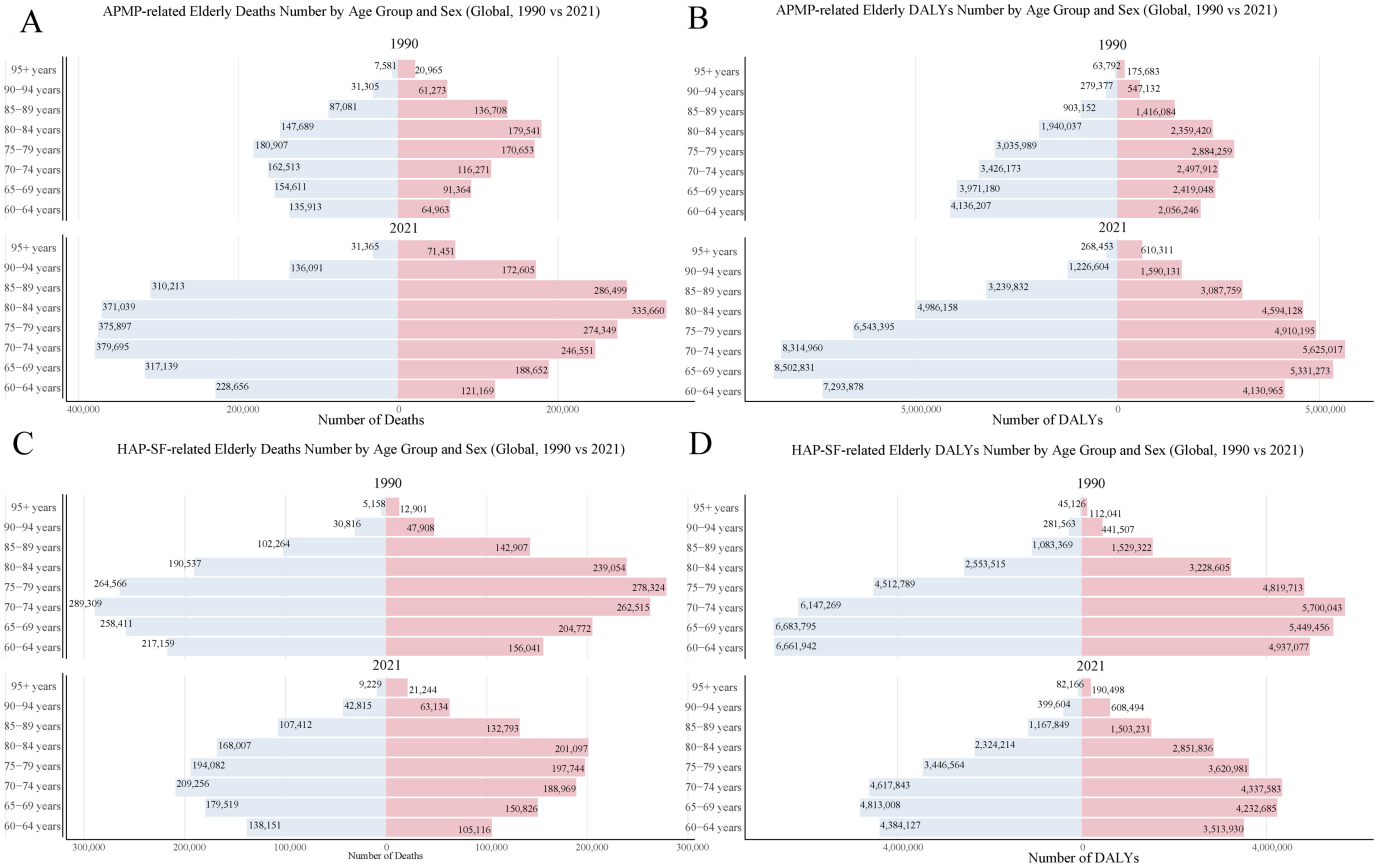
PMP-related older adult death and DALYs in 1990 and 2021 by sex and age group. **(A)** APMP-related older adult death number; **(B)** APMP-related older adult DALYs number; **(C)** HAP-SF-related older adult death number; **(D)** HAP-SF-related older adult DALYs number. PMP, particulate matter pollution; APMP, ambient particulate matter pollution; HAP-SF, household air pollution from solid fuels; DALYs, disability-adjusted life years.

From 1990 to 2021, the impact of PMP on older adult health underwent significant changes (Figures 1, 2). APMP-related older adult deaths increased from 1,750,000 to 3,850,000, while DALYs rose from 32,000,000 to 70,000,000 (Supplementary Tables S1, S2). However, the age-standardized mortality rate (ASMR) decreased from 384 to 337, and the age-standardized DALYs rate (ASDR) slightly declined from 6,817 to 6,309, indicating a reduction in relative risk.

Conversely, HAP-SF-related older adult deaths decreased from 2,700,000 to 2,100,000, with DALYs falling from 54,000,000 to 42,000,000 (Supplementary Tables S3, S4). The ASMR significantly dropped from 578 to 188, and the ASDR markedly decreased from 11,300 to 3,800, demonstrating a substantial reduction in the impact of HAP-SF on older adult health.

### Age and gender disparities

Mortality and DALYs rates due to PMP increased with age (Figure 1). In both 1990 and 2021, APMP-related older adult mortality and DALYs rates were generally higher in females than males, with this disparity being most pronounced in older age groups. In 2021, the highest number of APMP-related older adult deaths occurred in the 80–84 age group, totaling 710,000. Within this group, there were 370,000 female deaths compared to 335,660 male deaths (Figure 2A). Interestingly, the peak in DALYs occurred in a slightly younger age group. The 75–79 age group experienced the highest DALY count, totaling 10,000,000. The gender gap was even more pronounced here, with 6,500,000 DALYs in females compared to 4,900,000 in males (Figure 2B).

HAP-SF-related mortality and DALYs rates also increased with age (Figure 1). In 1990, females were significantly more affected by HAP-SF than males, but this gender gap had notably narrowed by 2021. In 1990, HAP-SF impact was most severe in the 75–79 age group, with 542,890 deaths (260,000 females, 280,000 males) and 9,300,000 DALYs (4,500,000 females, 4,800,000 males). By 2021, these figures had substantially decreased, with the 80–84 age group becoming the most affected by HAP-SF (Figures 2C,D).

### Geographical distribution and regional differences

**Figure 3:**
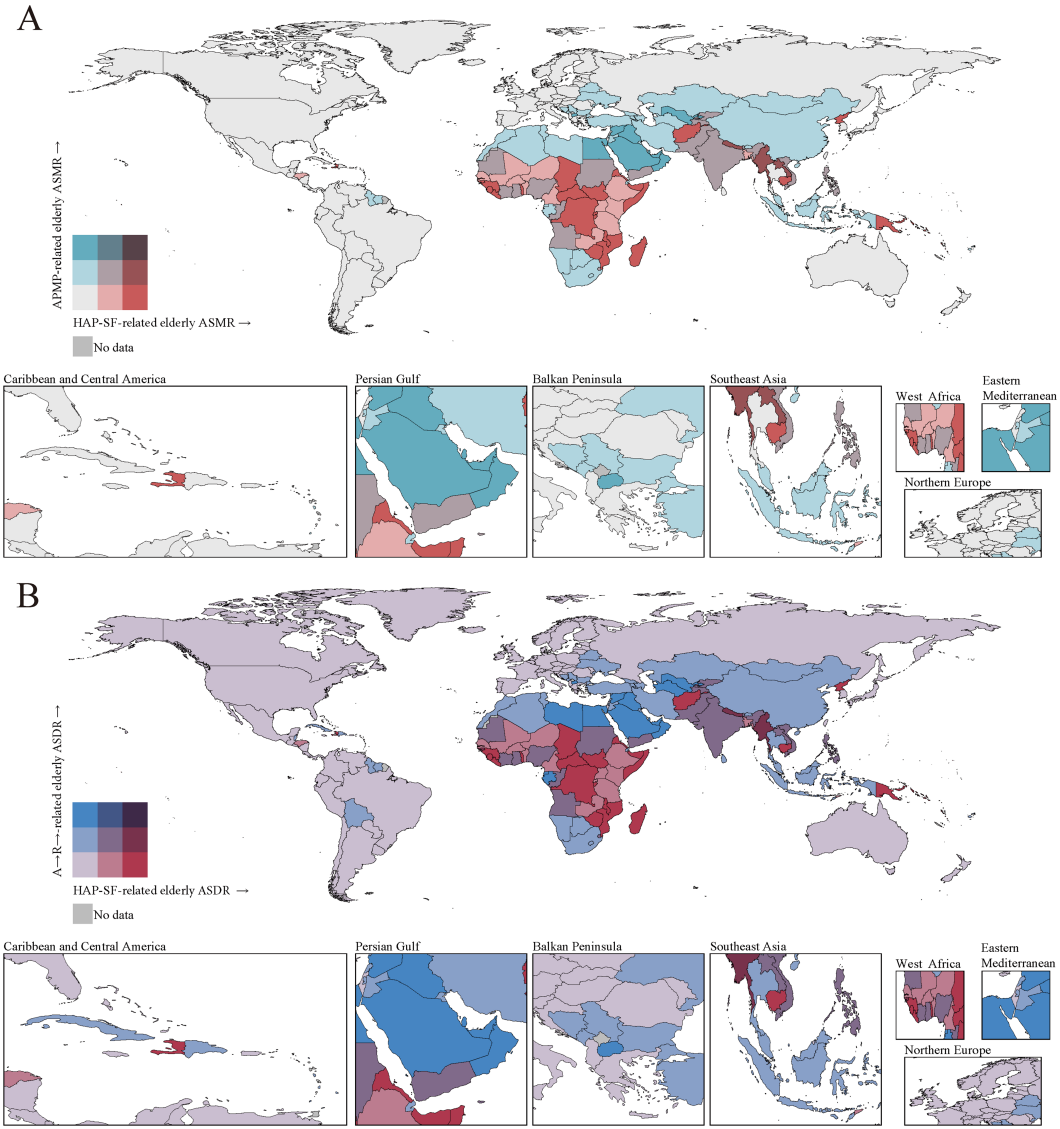
ASRs for PMP-related older adult deaths **(A)** and DALYs **(B)** in 2021 by country (or territory). ASMR, age-standardized mortality rate; ASDR, age-standardized disability-adjusted life-years rate; ASR, age-standardized rate; PMP, particulate matter pollution; APMP, ambient particulate matter pollution; HAP-SF, household air pollution from solid fuels.

High APMP-related older adult deaths and DALYs was primarily concentrated in Asia, Africa, and the Middle East (Figure 3A). In 2021, East Asia bore the heaviest burden, recording 1,680,000 APMP-related older adult deaths. This region not only had the highest absolute number of deaths but also a concerning ASMR of 619 per 100,000 older adult individuals. As shown in Supplementary Table S1, other regions with particularly high ASMRs included Central Asia, South Asia, North Africa, and the Middle East. The stark contrast becomes evident when we compare these figures to those of Western Europe and high-income North America. These regions exhibited significantly lower ASMRs of 70 and 46 per 100,000 older adult individuals, respectively—more than an order of magnitude lower than East Asia’s rate.

**Figure 4:**
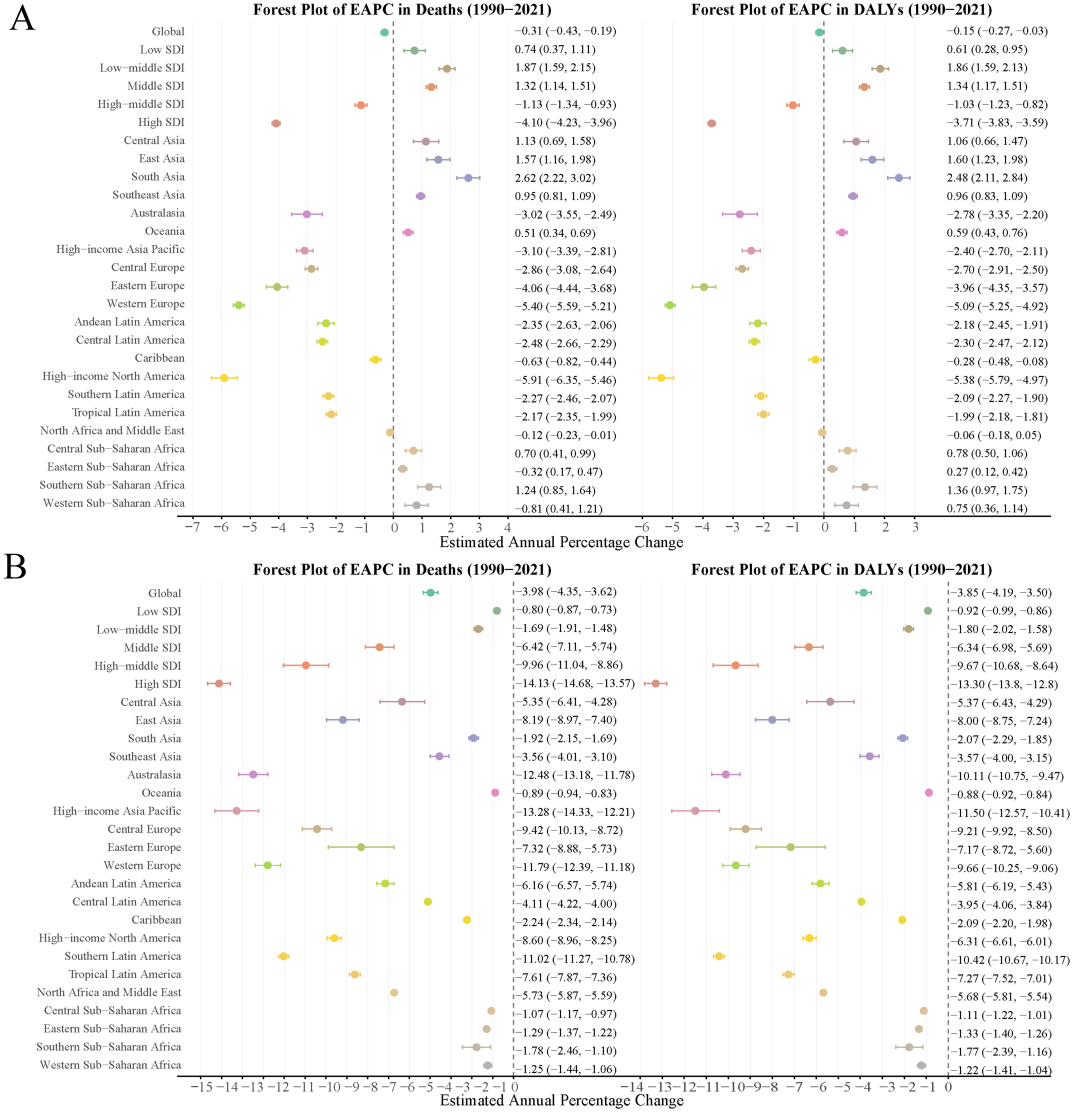
EAPCs of the ASRs for PMP-related older adult deaths and DALYs in Global and 26 regions. **(A)** EAPCs of the ASRs for APMP-related older adult deaths and DALYs; **(B)** EAPCs of the ASRs for HAP-SF-related older adult deaths and DALYs. ASR, age-standardized rate; PMP, particulate matter pollution; APMP, ambient particulate matter pollution; HAP-SF, household air pollution from solid fuels; DALYs, disability-adjusted life years; EAPC, estimated annual percentage change.

HAP-SF-related older adult deaths and DALYs was mainly concentrated in Central and Eastern Africa, parts of South Asia, and some Southeast Asian countries (Figure 3B). We find that South Asia bore the heaviest burden in absolute terms. In 2021, this region reported 1,020,000 HAP-SF-related older adult deaths, coupled with an alarmingly high age-standardized mortality rate (ASMR) of 616 per 100,000 older adult individuals. However, when examining ASMRs, several other regions emerge as areas of significant concern: 1. Oceania: Despite its smaller population, this region had the highest ASMR at 1,256 per 100,000 older adult individuals—more than double that of South Asia. 2. Central sub-Saharan Africa: With an ASMR of 1,016, this region closely followed Oceania in terms of relative risk. 3. Eastern sub-Saharan Africa: This region also showed a very high ASMR of 867. These figures starkly contrast with those from more developed regions. As evident from the pale areas in Figure 4B, Western Europe, high-income Asia-Pacific countries, and North America have reduced their HAP-SF burden to negligible levels. This dramatic difference underscores the strong link between HAP-SF exposure and socioeconomic development.

### Regional variations in trends

The EAPC in global APMP-related older adult deaths was −0.31 (95% CI: −0.43, −0.19), and −0.15 (95% CI: −0.27, −0.03) for DALYs, indicating a slight decrease in APMP-related older adult health burden. However, trend variations were significant across regions. South Asia, East Asia, and Central Asia showed increasing EAPCs, while Western Europe, high-income North America, and high-income Asia-Pacific exhibited significant decreasing trends. Low and low-middle Socio-demographic Index (SDI) regions demonstrated increasing EAPCs, whereas high SDI regions showed significant decreases (Figure 4A).

HAP-SF-related older adult deaths and DALYs displayed a more pronounced global decreasing trend. The EAPC for global HAP-SF-related older adult deaths was −3.98 (95% CI: −4.35, −3.62), and −3.85 (95% CI: −4.19, −3.50) for DALYs. Nearly all regions showed decreasing trends, with high SDI regions, Western Europe, and high-income Asia-Pacific experiencing the largest declines, with EAPC values below −10. However, Oceania and Central and Eastern sub-Saharan Africa showed relatively smaller declines, with EAPC values close to −1 (Figure 4B).

### Relationship between socioeconomic development and PMP-related health burden in the older adult

**Figure 5:**
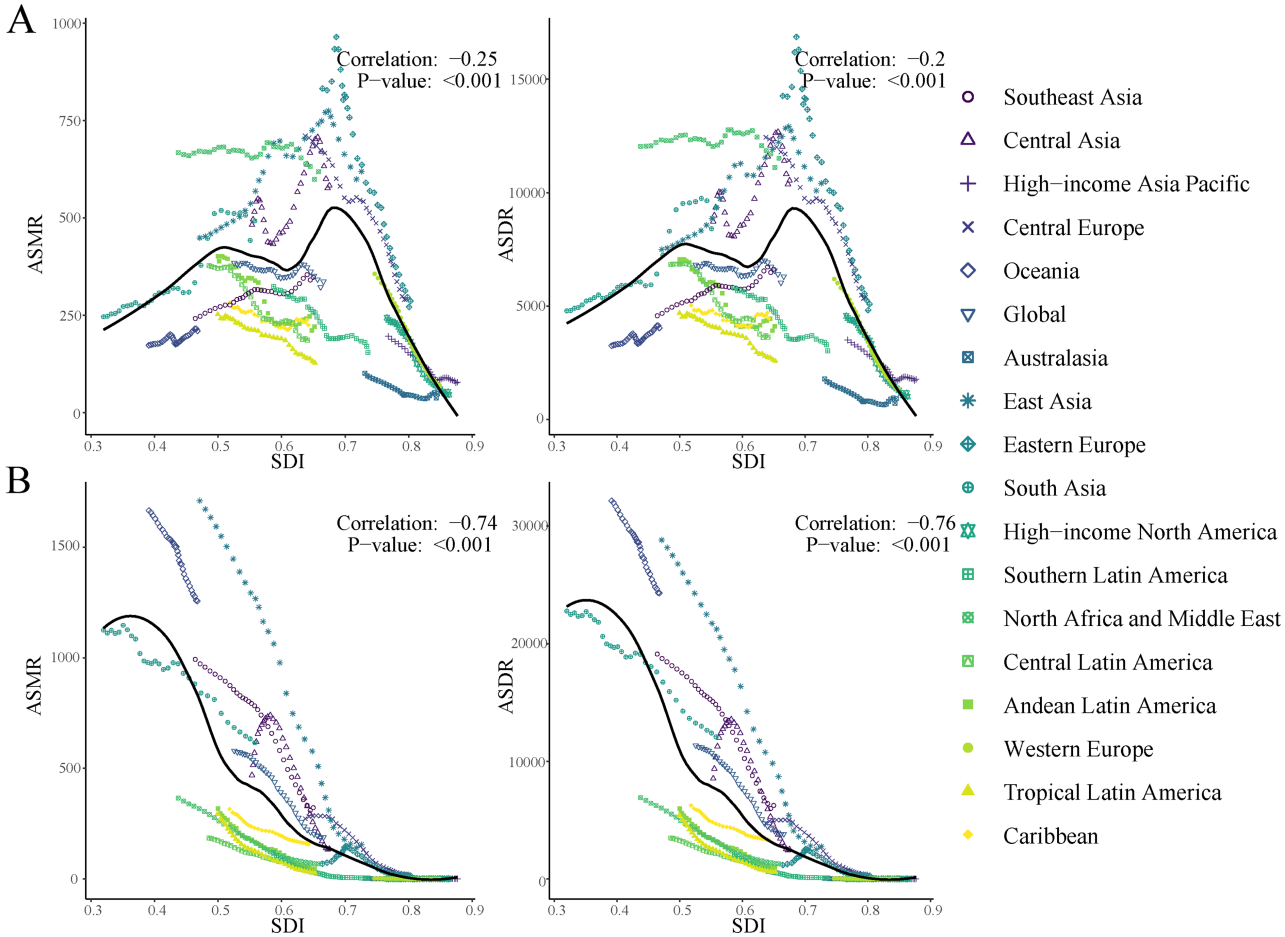
ASRs for PMP-related older adult death and DALYs in different GBD regions by SDI, 1990–2021. **(A)** ASRs for APMP-related older adult death and DALYs rate in different GBD regions by SDI; **(B)** ASRs for HAP-SF-related older adult death and DALYs in different GBD regions by SDI. ASMR, age-standardized mortality rate; ASDR, age-standardized disability-adjusted life-years rate; ASR, age-standardized rate; PMP, particulate matter pollution; APMP, ambient particulate matter pollution; HAP-SF, household air pollution from solid fuels.

Figures 5 and Supplementary Figure S1 illustrate the relationship between PMP-related older adult health burden in the older adult and the SDI from 1990 to 2021, providing in-depth analysis at both GBD region and country/territory levels. For APMP, GBD regional analysis showed a weak negative correlation with SDI (ASMR: *r* = −0.25, *p* < 0.001; ASDR: *r* = −0.27, *p* < 0.001), while country-level analysis revealed almost no correlation (ASMR: *r* = −0.04, *p* = 0.56; ASDR: *r* = −0.04, *p* = 0.54), reflecting that APMP is a ubiquitous issue across all development stages. In contrast, HAP-SF showed strong negative correlations at both regional (ASMR: *r* = −0.74, *p* < 0.001; ASDR: *r* = −0.76, *p* < 0.001) and country levels (both *r* = −0.83, *p* < 0.001), indicating that HAP-SF-related older adult health burden in the older adult significantly decreases as socioeconomic development levels improve.

## Discussion

This study provides a comprehensive analysis of global trends in the health impacts of PMP on the older adult from 1991 to 2021. Our findings both support and extend previous research on the impacts of PMP on older adult health. The increasing absolute numbers of PMP-related deaths and DALYs among the older adult from 1990 to 2021, despite decreasing age-standardized rates, aligns with global trends reported by Cohen et al. (7). However, our results show a more pronounced increase in APMP-related burden among the older adult compared to general population trends reported by Burnett et al. (2), likely reflecting the increased vulnerability of the older adult to air pollution.

Our analysis revealed a complex interplay between demographic shifts and air quality trends. We observed a significant increase in absolute numbers of APMP-related older adult deaths and DALYs, which aligns closely with global population aging trends (11). This increase in absolute burden underscores the growing public health challenge posed by APMP in an aging world. However, we also noted a slight decrease in age-standardized rates, suggesting a reduction in overall risk. This paradoxical finding likely reflects the positive outcomes of global air quality improvement measures, which have partially mitigated the increased vulnerability of a growing older adult population (12). Despite these improvements, it’s crucial to recognize that APMP remains a serious public health issue, particularly in rapidly urbanizing and industrializing regions (13). The persistent high burden in these areas highlights the need for targeted interventions that consider both demographic changes and economic development patterns. As urbanization continues globally, addressing APMP will require innovative strategies that balance economic growth with stringent air quality controls, especially in regions experiencing rapid industrial expansion.

The health burden attributed to HAP-SF showed a marked decreasing trend, likely due to the promotion of clean energy and improvements in indoor environments (14–16). However, HAP-SF continues to pose significant health threats in some low-and middle-income countries, especially in sub-Saharan Africa (17). Notably, indoor emissions escaping outdoors have become a major source of ambient fine particulate matter pollution in these regions (18). This phenomenon underscores the urgency of promoting clean energy use globally and highlights the close connection between indoor and outdoor air pollution. Addressing HAP-SF can thus improve indoor environments and significantly reduce outdoor air pollution, particularly in resource-limited areas.

Our study revealed significant age and gender disparities in the health impacts of APMP and HAP-SF. PMP-related mortality and DALYs rates increased with age, consistent with the heightened vulnerability of the older adult due to physiological decline and weakened immunity (6). Gender disparities, with females generally experiencing higher health burdens, stem from multiple factors. First, cultural practices and gender roles in many societies often result in women spending more time indoors, particularly in low-and middle-income countries. This increased exposure to household air pollution, especially from solid fuel use (HAP-SF), may partially explain the higher burden among women (19). For instance, in rural areas of South Asia and sub-Saharan Africa, women are typically responsible for cooking and household chores, leading to prolonged exposure to indoor air pollutants (20). Biological differences may also play a role. Some studies suggest that women might be more susceptible to the adverse effects of air pollution due to differences in lung anatomy and physiology, hormonal factors, and differential deposition of particulate matter in the lungs (21). For example, women tend to have smaller airways relative to lung size compared to men, which may increase their vulnerability to respiratory pollutants (22). These findings emphasize the need for gender-specific considerations in air pollution control strategies, such as targeted education programs for women, improved indoor cooking facilities, and stricter workplace air quality standards.

Our study revealed significant regional disparities in PMP-related health burdens among the older adult, with Asia and Africa bearing disproportionately high burdens compared to other regions. These disparities likely stem from a complex interplay of socioeconomic and environmental factors. In the case of APMP, the high burden observed in East Asia, South Asia, and parts of Africa can be attributed to rapid industrialization and urbanization, often occurring without adequate environmental regulations or enforcement (23). Many cities in these regions face severe air pollution due to industrial emissions, vehicular exhaust, and construction activities. Additionally, geographical and meteorological factors, such as dust storms in North Africa and Eastern Asia, exacerbate the problem in certain areas (24).

The high HAP-SF burden in Central and Eastern Africa, parts of South Asia, and some Southeast Asian countries is closely linked to poverty and lack of access to clean cooking fuels and technologies (25, 26). In contrast, Western Europe, high-income Asia-Pacific countries, and North America have significantly lower burdens of both APMP and HAP-SF, benefiting from decades of stringent air quality regulations, advanced pollution control technologies, and widespread access to clean cooking fuels (27). These regional disparities underscore the need for targeted policy interventions, including stricter emission standards and promotion of cleaner technologies for APMP, and expanded access to clean cooking fuels for HAP-SF. Notably, the lack of correlation between APMP-related health burden and SDI indicates that APMP is a global issue requiring concerted efforts from all countries. In contrast, the strong negative correlation between HAP-SF and SDI underscores the importance of promoting socioeconomic development to improve indoor air quality (28).

Our findings provide crucial evidence for developing targeted air quality improvement strategies, highlighting the need for differentiated approaches to address APMP and HAP-SF. For APMP, which shows no significant correlation with socioeconomic development, there is a need to strengthen regional cooperation to address transboundary pollution issues. This approach should be coupled with enhanced environmental regulation and clean technology application in rapidly urbanizing areas, regardless of their development status (29). In contrast, HAP-SF, which demonstrates a strong negative correlation with socioeconomic development, requires continued promotion of clean energy use, particularly in low-and middle-income countries (30). This stark difference in the relationship between these two types of pollution and socioeconomic development underscores the complexity of addressing PMP globally and the need for context-specific interventions.

This study has several limitations. First, due to data availability constraints, we were unable to analyze changes at finer geographical scales or shorter time intervals. Second, our focus on mortality and DALY indicators may not fully capture the comprehensive impact of particulate matter pollution on older adult quality of life. Future research should consider incorporating additional health indicators, such as hospitalization rates and chronic disease incidence, to provide a more comprehensive assessment. Furthermore, this study did not explore in depth the interactive effects of particulate matter pollution and other environmental factors (e.g., temperature, humidity) on older adult health, which represents an important direction for future research (31).

Our study provides valuable insights into long-term trends of particulate matter pollution’s impact on global older adult health. Our results emphasize the importance of continued air quality improvement, especially in the context of population aging challenges. Moving forward, increased interdisciplinary and intersectoral collaboration is necessary to develop more effective policies and interventions to protect older adult health and achieve sustainable development goals. These efforts should include targeted clean cooking initiatives to reduce HAP-SF exposure, particularly in areas where women are primarily responsible for cooking. Simultaneously, health education for the older adult should be strengthened to enhance their awareness of air pollution risks and self-protection capabilities, with a focus on gender-specific programs that address women’s higher vulnerability to air pollution. Additionally, urban planning and public health policy formulation should specifically consider the needs of the older adult, such as increasing green space and optimizing public transportation systems, to reduce their exposure to particulate matter pollution. This gender-responsive urban planning approach should ensure that public spaces frequented by older adult women have adequate air pollution mitigation measures. Furthermore, we recommend integrating gender considerations into national and local air quality policies to address the specific needs and vulnerabilities of older adult women. Finally, encouraging more research on the biological mechanisms underlying gender differences in air pollution susceptibility could inform more targeted medical interventions and prevention strategies, ultimately leading to more equitable and effective approaches to reduce PMP-related health burdens among the older adult population.

In conclusion, addressing particulate matter pollution is not just an environmental issue, but a matter of intergenerational justice and sustainable development. As we face an increasingly aged global population, the choices we make today will determine the health and well-being of generations to come. This study serves as a call to action for more research, stronger policies, and a collective commitment to ensuring clean air and healthy aging for all.

## Data Availability

Publicly available datasets were analyzed in this study. This data can be found at: https://vizhub.healthdata.org/gbd-results/.

## References

[1] Schraufnagel D Balmes J Cowl C De Matteis S Jung S Mortimer K. Air pollution and noncommunicable diseases: a review by the forum of international respiratory Societies' environmental committee, part 1: the damaging effects of air pollution. Chest. (2019) 155:409–16. doi: 10.1016/j.chest.2018.10.042, PMID: 30419235 PMC6904855

[2] Burnett R Chen H Szyszkowicz M Fann N Hubbell B Pope C. Global estimates of mortality associated with long-term exposure to outdoor fine particulate matter. Proc Natl Acad Sci USA. (2018) 115:9592–7. doi: 10.1073/pnas.1803222115, PMID: 30181279 PMC6156628

[3] Matteo B Pietro S Marina C Riccardo B. Assessment of indoor-outdoor particulate matter air pollution: a review. Atmosphere. (2017) 8:136. doi: 10.3390/atmos8080136

[4] India State-Level Disease Burden Initiative Air Pollution Collaborators. The impact of air pollution on deaths, disease burden, and life expectancy across the states of India: the global burden of disease study 2017. Lancet Planet Health. (2019) 3:e26–39. doi: 10.1016/S2542-5196(18)30261-4 30528905 PMC6358127

[5] Simoni M Baldacci S Maio S Cerrai S Sarno G Viegi G. Adverse effects of outdoor pollution in the elderly. J Thorac Dis. (2015) 7:34–45. doi: 10.3978/j.issn.2072-1439.2014.12.10, PMID: 25694816 PMC4311079

[6] Bell M Zanobetti A Dominici F. Evidence on vulnerability and susceptibility to health risks associated with short-term exposure to particulate matter: a systematic review and meta-analysis. Am J Epidemiol. (2013) 178:865–76. doi: 10.1093/aje/kwt090, PMID: 23887042 PMC3775545

[7] Cohen A Brauer M Burnett R Anderson H Frostad J Estep K. Estimates and 25-year trends of the global burden of disease attributable to ambient air pollution: an analysis of data from the global burden of diseases study 2015. Lancet. (2017) 389:1907–18. doi: 10.1016/S0140-6736(17)30505-6, PMID: 28408086 PMC5439030

[8] Yin P Brauer M Cohen A Wang H Li J Burnett R. The effect of air pollution on deaths, disease burden, and life expectancy across China and its provinces, 1990-2017: an analysis for the global burden of disease study 2017. Lancet Planet Health. (2020) 4:e386–98. doi: 10.1016/S2542-5196(20)30161-3, PMID: 32818429 PMC7487771

[9] GBD 2017 Risk Factor Collaborators. Global, regional, and national comparative risk assessment of 84 behavioural, environmental and occupational, and metabolic risks or clusters of risks for 195 countries and territories, 1990-2017: a systematic analysis for the global burden of disease study 2017. Lancet. (2018) 392:1923–94. doi: 10.1016/S0140-6736(18)32225-6 30496105 PMC6227755

[10] Li J Liu H Lv Z Zhao R Deng F Wang C. Estimation of PM 2.5 mortality burden in China with new exposure estimation and local concentration-response function. Environ Pollut. (2018) 243:1710–8. doi: 10.1016/j.envpol.2018.09.089, PMID: 30408858

[11] Elbarbary M Honda T Morgan G Guo Y Guo Y Kowal P. Ambient air pollution exposure association with anaemia prevalence and haemoglobin levels in Chinese older adults. Int J Environ Res Public Health. (2020) 17:3209. doi: 10.3390/ijerph17093209, PMID: 32380747 PMC7246731

[12] Zhang Z Wang J Kwong J Burnett R van Donkelaar A Hystad P. Long-term exposure to air pollution and mortality in a prospective cohort: the Ontario health study. Environ Int. (2021) 154:106570. doi: 10.1016/j.envint.2021.106570, PMID: 33892223

[13] Liu C Chen R Sera F Vicedo-Cabrera A Guo Y Tong S. Ambient particulate air pollution and daily mortality in 652 cities. N Engl J Med. (2019) 381:705–15. doi: 10.1056/NEJMoa1817364, PMID: 31433918 PMC7891185

[14] Shao Y Liu R Yang J Liu M Fang W Hu L. Economic growth facilitates household fuel use transition to reduce PM 2.5-related deaths in China. Environ Sci Technol. (2023) 57:12663–73. doi: 10.1021/acs.est.3c03276, PMID: 37558636

[15] Zhao B Zheng H Wang S Smith K Lu X Aunan K. Change in household fuels dominates the decrease in PM 2.5 exposure and premature mortality in China in 2005-2015. Proc Natl Acad Sci USA. (2018) 115:12401–6. doi: 10.1073/pnas.1812955115, PMID: 30455309 PMC6298076

[16] Zheng H Zhao B Wang S Wang T Ding D Chang X. Transition in source contributions of PM 2.5 exposure and associated premature mortality in China during 2005-2015. Environ Int. (2019) 132:105111. doi: 10.1016/j.envint.2019.105111, PMID: 31476640

[17] Kodros J Carter E Brauer M Volckens J Bilsback K L'Orange C. Quantifying the contribution to uncertainty in mortality attributed to household, ambient, and joint exposure to PM 2.5 from residential solid fuel use. Geohealth. (2018) 2:25–39. doi: 10.1002/2017GH000115, PMID: 32158998 PMC7007171

[18] Chowdhury S Pillarisetti A Oberholzer A Jetter J Mitchell J Cappuccilli E. A global review of the state of the evidence of household air pollution's contribution to ambient fine particulate matter and their related health impacts. Environ Int. (2023) 173:107835. doi: 10.1016/j.envint.2023.107835, PMID: 36857905 PMC10378453

[19] Sukhsohale N Narlawar U Phatak M. Indoor air pollution from biomass combustion and its adverse health effects in Central India: an exposure-response study. Indian J Community Med. (2013) 38:162–7. doi: 10.4103/0970-0218.116353, PMID: 24019602 PMC3760325

[20] Amegah A Jaakkola J. Household air pollution and the sustainable development goals. Bull World Health Organ. (2016) 94:215–21. doi: 10.2471/BLT.15.155812, PMID: 26966333 PMC4773927

[21] Kim H Noh J Noh Y Oh S Koh S Kim C. Gender difference in the effects of outdoor air pollution on cognitive function among elderly in Korea. Front Public Health. (2019) 7:375. doi: 10.3389/fpubh.2019.00375 31921740 PMC6915851

[22] Clougherty J. A growing role for gender analysis in air pollution epidemiology. Environ Health Perspect. (2010) 118:167–76. doi: 10.1289/ehp.0900994, PMID: 20123621 PMC2831913

[23] Park J Kim H Kim Y Heo J Kim S Jeon K. Source apportionment of PM 2.5 in Seoul, South Korea and Beijing, China using dispersion normalized PMF. Sci Total Environ. (2022) 833:155056. doi: 10.1016/j.scitotenv.2022.155056, PMID: 35395292

[24] Goudie A. Desert dust and human health disorders. Environ Int. (2014) 63:101–13. doi: 10.1016/j.envint.2013.10.011 24275707

[25] Bonjour S Adair-Rohani H Wolf J Bruce N Mehta S Prüss-Ustün A. Solid fuel use for household cooking: country and regional estimates for 1980–2010. Environ Health Perspect. (2013) 121:784–90. doi: 10.1289/ehp.1205987, PMID: 23674502 PMC3701999

[26] Smith K Bruce N Balakrishnan K Adair-Rohani H Balmes J Chafe Z. Millions dead: how do we know and what does it mean? Methods used in the comparative risk assessment of household air pollution. Annu Rev Public Health. (2014) 35:185–206. doi: 10.1146/annurev-publhealth-032013-182356, PMID: 24641558

[27] Lelieveld J Evans J Fnais M Giannadaki D Pozzer A. The contribution of outdoor air pollution sources to premature mortality on a global scale. Nature. (2015) 525:367–71. doi: 10.1038/nature15371, PMID: 26381985

[28] Landrigan P Fuller R Acosta N Adeyi O Arnold R Basu N. The lancet commission on pollution and health. Lancet. (2018) 391:462–512. doi: 10.1016/S0140-6736(17)32345-0 29056410

[29] Vohra K Vodonos A Schwartz J Marais E Sulprizio M Mickley L. Global mortality from outdoor fine particle pollution generated by fossil fuel combustion: results from GEOS-chem. Environ Res. (2021) 195:110754. doi: 10.1016/j.envres.2021.110754, PMID: 33577774

[30] Sambandam S Balakrishnan K Ghosh S Sadasivam A Madhav S Ramasamy R. Can currently available advanced combustion biomass cook-stoves provide health relevant exposure reductions? Results from initial assessment of select commercial models in India. EcoHealth. (2015) 12:25–41. doi: 10.1007/s10393-014-0976-1, PMID: 25293811

[31] Chen K Wolf K Breitner S Gasparrini A Stafoggia M Samoli E. Two-way effect modifications of air pollution and air temperature on total natural and cardiovascular mortality in eight European urban areas. Environ Int. (2018) 116:186–96. doi: 10.1016/j.envint.2018.04.021, PMID: 29689465

