# Supplementary material for: Particulate matter pollution and older adult health: global trends and disparities, 1991–2021
Source: Front Public Health. 2024 Nov 6;12:1478860. doi: 10.3389/fpubh.2024.1478860 (PMC11576382; doi:10.3389/fpubh.2024.1478860)
Supplement: Supplementary file 1 [file Data_Sheet_1.docx]

## **
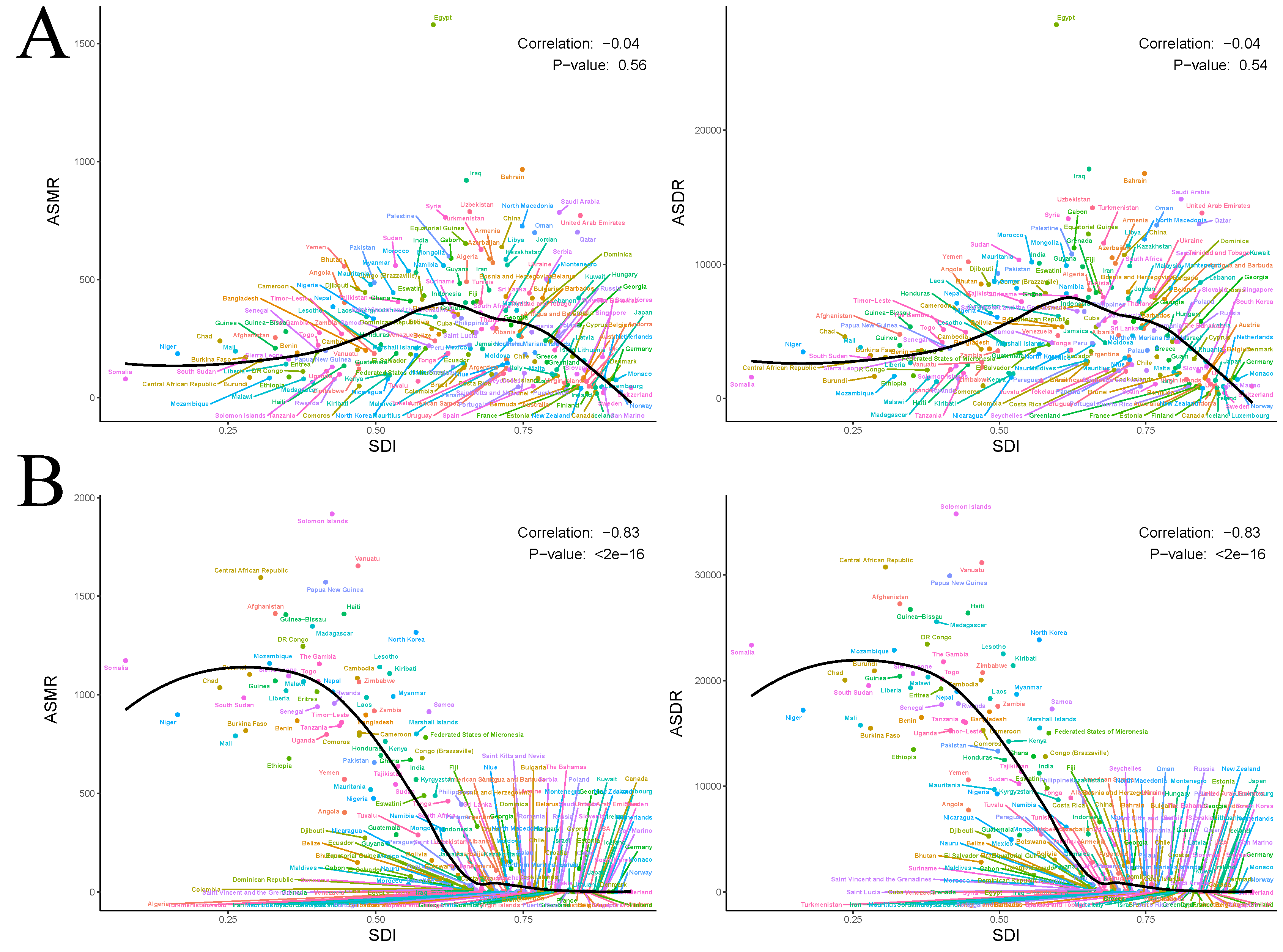
**

**Supplementary Fiqure 1.** ASRs for PMP-related elderly deaths and DALYs in 204 countries and territories by SDI, 1990-2021.  (A) ASRs for APMP-related elderly deaths and DALYs rate in 204 countries and territories by SDI; (B) ASRs for HAP-SF-related elderly deaths and DALYs in 204 countries and territories by SDI.

ASMR, age-standardized mortality rate; ASDR, age-standardized disability-adjusted life-years rate; ASR, age-standardized rate; PMP, particulate matter pollution; APMP, ambient particulate matter pollution; HAP-SF, household air pollution from solid fuels.

## **Supplementary Table 1.** APMP-related elderly deaths and ASMR in 1990 and 2021.

|  | **1990** | | **2021** | |
| --- | --- | --- | --- | --- |
| **Characteristics** | **Death cases** | **ASMR** | **Death cases** | **ASMR** |
|  | **No. ×10^3^ (95% UI)** | **(95% UI)** | **No. ×10^3^ (95% UI)** | **(95% UI)** |
| Overall | 1749.34 (1235.88-2269.16) | 384.25 (271.03-499.24) | 3847.03 (2820.65-4718.49) | 336.54 (246.75-412.5) |
| Sex |  |  |  |  |
| Male | 907.6 (650.75-1169.5) | 481.71 (344.73-621.86) | 2150.09 (1613.39-2625.06) | 438.39 (329.11-535.11) |
| Female | 841.74 (590.25-1097.16) | 311.94 (218.51-406.99) | 1696.94 (1186.94-2141.23) | 257.36 (180.56-324.52) |
| Socio-demographic index |  |  |  |  |
| Low | 47.07 (30.56-67.66) | 215.58 (139.27-310.51) | 117.86 (78.17-166.94) | 237.1 (156.91-335.9) |
| Low-middle | 160.07 (105.78-228.89) | 260.51 (171.94-371.91) | 665.46 (419.46-919.2) | 414.88 (260.97-573.2) |
| Middle | 376.95 (245.76-541.83) | 371.13 (242.37-531.96) | 1619.72 (1113.86-1976.53) | 504.87 (346.99-616.32) |
| Middle-high | 656.42 (454.93-879.08) | 569.88 (394.12-763.6) | 1098.62 (845.66-1346.53) | 397.75 (306.6-487.29) |
| High | 505.95 (327.69-713.34) | 319.99 (207.73-450.67) | 342.5 (245.88-447.28) | 96.65 (70.73-125.19) |
| Region |  |  |  |  |
| Central Asia | 26.53 (10.47-49.15) | 482.11 (190.43-893.24) | 50.91 (35.04-65.65) | 582.93 (400.92-751.88) |
| East Asia | 356.31 (170.35-625.91) | 448.55 (212.7-789.87) | 1681.92 (1172.47-2089.3) | 619.07 (431.48-769.23) |
| South Asia | 138.99 (74.82-222) | 246.08 (132.22-393.6) | 813.63 (520.27-1094.98) | 492.2 (314.13-662.05) |
| Southeast Asia | 62.54 (28.28-110.68) | 241.37 (109.04-427.9) | 249.49 (158.42-334.38) | 347.43 (220.39-465.34) |
| Australasia | 3.26 (0.12-8.92) | 100.86 (3.57-275.5) | 4.51 (2.66-6.7) | 48.27 (28.56-71.28) |
| Oceania | 0.44 (0.13-1.05) | 172.74 (51.6-404.12) | 1.41 (0.5-3.02) | 209.78 (74.48-447.01) |
| High-income Asia Pacific | 49.26 (13.69-97.77) | 195.82 (54.27-389.02) | 72.22 (41.52-106.57) | 78.44 (46.37-113.18) |
| Central Europe | 132.94 (69.07-202.4) | 710.29 (368.82-1081.78) | 101.2 (78.8-125.48) | 290.81 (227.49-360.17) |
| Eastern Europe | 287.9 (148.25-430.8) | 832.18 (427.79-1246.77) | 147.74 (92.96-217.44) | 286.12 (180.5-421.1) |
| Western Europe | 308.84 (157.19-490.03) | 356.14 (181.05-564.33) | 121.08 (83.83-163.02) | 69.86 (48.79-93.48) |
| Andean Latin America | 9.6 (4.54-15.47) | 400.95 (189.81-645.53) | 17.38 (10.29-25.71) | 220.51 (131.26-325.86) |
| Central Latin America | 34.73 (19.22-51.42) | 377.09 (208.43-558.7) | 60.86 (42.44-80.28) | 186.02 (130.01-245.34) |
| Caribbean | 9.14 (3.54-17.04) | 282.47 (109.36-526.64) | 16.99 (8.8-26.65) | 224.75 (116.93-352.89) |
| High-income North America | 127.62 (51.64-219.82) | 243.74 (98.66-418.99) | 47.8 (22.91-76.87) | 45.85 (22.1-73.36) |
| Southern Latin America | 19.02 (9.55-31.68) | 323.66 (162.26-539.29) | 20.19 (11.09-30.2) | 154.54 (85.65-230.31) |
| Tropical Latin America | 24.92 (8.91-46.96) | 252.16 (89.87-476.61) | 43.16 (24.05-64.37) | 127.58 (71.42-189.81) |
| North Africa and Middle East | 112.55 (82.25-140.29) | 667.25 (487.08-832.01) | 295.13 (237.91-350.86) | 618.33 (497.95-734.8) |
| Central Sub-Saharan Africa | 3.6 (1.93-5.85) | 185.68 (100-300.41) | 9.66 (5.77-14.59) | 204.55 (122.33-309.01) |
| Eastern Sub-Saharan Africa | 7.38 (4.88-10.82) | 101.21 (66.52-148.81) | 16.02 (9.88-24.25) | 99.07 (60.78-150.25) |
| Southern Sub-Saharan Africa | 7.68 (5.05-10.35) | 258.22 (169.54-348.23) | 20.83 (14.57-27.06) | 337.02 (234.9-439.06) |
| Western Sub-Saharan Africa | 26.08 (15.32-38.58) | 288.46 (168.86-429.03) | 54.92 (31.38-85.23) | 293.38 (167.71-454.11) |

APMP, ambient particulate matter pollution; ASMR, age-standardized mortality rate; UI, uncertainty interval.

## **Supplementary Table 2.** APMP-related elderly DALYs and ASDR in 1990 and 2021.

|  | **1990** | | **2021** | |
| --- | --- | --- | --- | --- |
| **Characteristics** | **DALYs** | **ASDR** | **DALYs** | **ASDR** |
|  | **No. ×10^3^ (95% UI)** | **(95% UI)** | **No. ×10^3^ (95% UI)** | **(95% UI)** |
| Overall | 32111.69 (22811.44-41530.56) | 6816.58 (4837.86-8825.65) | 70255.89 (51380.01-85754.42) | 6308.81 (4612.7-7697.78) |
| Sex |  |  |  |  |
| Male | 17755.91 (12799.59-22818.03) | 8705.79 (6266.54-11203.76) | 40376.11 (30237.43-49359.71) | 8099.78 (6068.56-9898.43) |
| Female | 14355.78 (10162.55-18585.8) | 5320.29 (3764.09-6891.18) | 29879.78 (21041.78-37234.24) | 4823.47 (3399.32-6006.36) |
| Socio-demographic index |  |  |  |  |
| Low | 986.86 (643.65-1415.05) | 4105.77 (2670.87-5893.04) | 2369.78 (1574.88-3339.34) | 4435.97 (2943.91-6251.15) |
| Low-middle | 3238.76 (2134.1-4647.11) | 4909.15 (3231.62-7035.57) | 13125.86 (8254.84-18079.41) | 7884.53 (4950.79-10860.17) |
| Middle | 7336.06 (4804.75-10475.42) | 6613.3 (4330.7-9436.54) | 29732.3 (20501.88-36292.81) | 9113.15 (6281.19-11123.84) |
| Middle-high | 11790.87 (8194.5-15790.12) | 9780.92 (6789.27-13103.88) | 18924.8 (14804.24-22958.77) | 7101.96 (5559.8-8611.29) |
| High | 8707.49 (5757.57-12159.07) | 5742.52 (3806.65-8009.45) | 6051.5 (4437.84-7817.87) | 1968.27 (1457.51-2531.16) |
| Region |  |  |  |  |
| Central Asia | 483.33 (193.25-890.77) | 8727.77 (3490.82-16096.79) | 961.37 (670.74-1227.55) | 10535.91 (7343.45-13460.59) |
| East Asia | 6787.64 (3264.44-11852.37) | 7499.62 (3587.2-13120.33) | 28813.82 (20015.68-35520.83) | 10411.43 (7236.49-12837.48) |
| South Asia | 2929.02 (1575.79-4675.7) | 4791.05 (2574.93-7654.64) | 16162.95 (10341.95-21608.89) | 9358.58 (5980.43-12507.05) |
| Southeast Asia | 1260.92 (577.06-2234.27) | 4574.23 (2091.18-8110.73) | 4994.69 (3217.24-6671.54) | 6625.79 (4267.91-8846.46) |
| Australasia | 57.03 (2.01-154.28) | 1789.42 (63.15-4840.64) | 75.18 (44.5-110.05) | 928.03 (550.01-1355.21) |
| Oceania | 9.59 (2.85-22.86) | 3255.63 (969.73-7683.88) | 30.01 (10.48-64.72) | 4039.14 (1420.82-8671.02) |
| High-income Asia Pacific | 889.77 (256.13-1722.51) | 3507.7 (1008.72-6792.77) | 1276.75 (784.52-1808.36) | 1779.1 (1117.77-2475.02) |
| Central Europe | 2383.43 (1246.77-3621.93) | 12435.57 (6501.76-18901.6) | 1739.78 (1357.09-2137.08) | 5383.49 (4203.97-6606.75) |
| Eastern Europe | 5082.94 (2628.77-7584.33) | 14258.39 (7366.6-21288.44) | 2520.23 (1591.76-3681.67) | 5087.96 (3219.95-7430.31) |
| Western Europe | 5092.83 (2603.96-7979.3) | 6197.08 (3166.92-9690.54) | 1949.77 (1368.91-2596.56) | 1355.26 (956.13-1799.2) |
| Andean Latin America | 167.4 (80.17-266.74) | 7054.02 (3379.81-11235) | 308.57 (190.88-444.54) | 4112.93 (2554.08-5917.86) |
| Central Latin America | 643.45 (355.31-944.75) | 6839.54 (3774.06-10049.42) | 1147.02 (797.18-1519.15) | 3634.66 (2527.43-4815) |
| Caribbean | 162.86 (63.35-299.56) | 5032.29 (1956.03-9253.05) | 313.19 (163.1-480.66) | 4453.85 (2325.22-6832.4) |
| High-income North America | 2245.18 (910.38-3815.76) | 4569.92 (1850.46-7749.99) | 956.21 (461.9-1544.6) | 1013.08 (490.39-1633.12) |
| Southern Latin America | 342.47 (173.79-566.77) | 5796.01 (2939.65-9593.26) | 364.03 (208.13-533.39) | 3029.44 (1740.28-4424.95) |
| Tropical Latin America | 486.81 (175.52-905.48) | 4688.71 (1686.96-8739.16) | 844.15 (482.99-1250.07) | 2576.14 (1477.05-3809.98) |
| North Africa and Middle East | 2185.96 (1601.83-2716.75) | 12036.71 (8816.67-14960.4) | 5749.83 (4684.25-6799.28) | 11514.57 (9380.16-13611.17) |
| Central Sub-Saharan Africa | 77.78 (41.65-126.11) | 3475.52 (1869.1-5619.29) | 205.52 (123.18-308.96) | 3899.4 (2341.87-5857.6) |
| Eastern Sub-Saharan Africa | 155.96 (103.28-227.35) | 1952.87 (1289.27-2852.38) | 328.03 (205.08-493.71) | 1889.01 (1177.99-2845.31) |
| Southern Sub-Saharan Africa | 149.59 (98.63-199.62) | 4862.56 (3206.14-6490.79) | 432.91 (305.46-557.84) | 6596.41 (4646.66-8512.87) |
| Western Sub-Saharan Africa | 517.72 (306.32-759.68) | 5357.44 (3163.12-7890.77) | 1081.89 (618.16-1668.12) | 5398.91 (3085.47-8307.96) |

APMP, ambient particulate matter pollution; DALYs, disability-adjusted life years; ASDR, age standardized DALYs rate; UI, uncertainty interval.

## **Supplementary Table 3.** HAP-SF-related elderly deaths and ASMR in 1990 and 2021.

|  | **1990** | | **2021** | |
| --- | --- | --- | --- | --- |
| **Characteristics** | **Death cases** | **ASMR** | **Death cases** | **ASMR** |
|  | **No. ×10^3^ (95% UI)** | **(95% UI)** | **No. ×10^3^ (95% UI)** | **(95% UI)** |
| Overall | 2702.64 (2165.65-3288.64) | 579.58 (463.4-706.59) | 2109.39 (1193.05-3781.52) | 187.79 (106.79-334.88) |
| Sex |  |  |  |  |
| Male | 1358.22 (1056.9-1660.41) | 694.78 (538.57-851.89) | 1048.47 (581.04-1951.65) | 212.08 (117.21-395.87) |
| Female | 1344.42 (1061.74-1665.07) | 497.23 (392.65-615.87) | 1060.92 (604.79-1844.43) | 168.42 (97.28-289.2) |
| Socio-demographic index |  |  |  |  |
| Low | 270.83 (220.76-319.04) | 1231.66 (996.42-1458.05) | 478.63 (382.63-573.59) | 960.1 (763.81-1151.8) |
| Low-middle | 666.37 (541.5-780.64) | 1072.91 (869.16-1261.79) | 1021.34 (654.93-1431.91) | 638.76 (409.3-895.35) |
| Middle | 1132.61 (914.37-1349.54) | 1100.35 (886.13-1313.15) | 522.11 (122.42-1354.69) | 162.94 (38.12-422.93) |
| Middle-high | 586.54 (406.84-825.41) | 499.76 (345.9-706.13) | 84.32 (5.23-450.3) | 30.69 (1.91-163.63) |
| High | 44.39 (14.51-106.17) | 28.42 (9.3-68.01) | 1.66 (0-15.75) | 0.47 (0-4.53) |
| Region |  |  |  |  |
| Central Asia | 25.77 (11.66-47.2) | 468.96 (212.15-858.89) | 11.82 (4.86-26.81) | 135.35 (55.29-308.06) |
| East Asia | 1363.02 (1065.08-1637.89) | 1710.59 (1335.81-2049.71) | 422.97 (104.62-1268.11) | 155.3 (38.34-465.53) |
| South Asia | 635.17 (514.1-747.79) | 1125.09 (906.97-1331.51) | 1018.27 (658.07-1450.8) | 615.76 (396.84-878.3) |
| Southeast Asia | 256.54 (202.58-308.83) | 992.83 (781.97-1197.35) | 230.49 (102.6-408.54) | 321.87 (143.28-569.91) |
| Australasia | 0.06 (0-0.6) | 1.71 (0-18.65) | 0 (0-0.02) | 0.04 (0-0.25) |
| Oceania | 4.31 (3.27-5.41) | 1666.58 (1269.06-2083.1) | 8.43 (6.17-10.66) | 1256.46 (920.54-1589.68) |
| High-income Asia Pacific | 0.8 (0.05-4.83) | 3.15 (0.22-19.17) | 0.03 (0-0.25) | 0.03 (0-0.26) |
| Central Europe | 50.52 (12.33-133.33) | 271.63 (66.59-714.24) | 7.72 (0.26-50.22) | 22.2 (0.76-144.02) |
| Eastern Europe | 23.42 (5.11-90.96) | 68.1 (14.86-263.65) | 7.42 (1.08-31.01) | 14.45 (2.13-60.06) |
| Western Europe | 1.62 (0.02-13.06) | 1.86 (0.02-15.02) | 0.08 (0-0.72) | 0.04 (0-0.41) |
| Andean Latin America | 7.62 (3.51-12.5) | 318.48 (147.26-521.72) | 3.56 (0.62-11.18) | 45.81 (8.15-142.75) |
| Central Latin America | 17.01 (6.87-32.98) | 185.36 (75.01-358.88) | 17.32 (5.75-44.13) | 53.37 (17.97-134.95) |
| Caribbean | 10.15 (7.01-13.8) | 316.07 (218.02-430.18) | 10.88 (7.59-15.28) | 156.36 (109.53-216.94) |
| High-income North America | 0.09 (0-0.76) | 0.18 (0-1.44) | 0.02 (0-0.09) | 0.02 (0-0.09) |
| Southern Latin America | 7.01 (1.81-17.63) | 119.27 (30.87-299.98) | 0.49 (0-4.62) | 3.7 (0.02-34.86) |
| Tropical Latin America | 28.46 (14.51-48.88) | 294.51 (150.63-503) | 9.27 (2.11-26.57) | 27.28 (6.24-78.24) |
| North Africa and Middle East | 62.55 (39.76-99.55) | 365.31 (230.16-586.46) | 32.38 (21.86-47.41) | 67.66 (45.67-99.15) |
| Central Sub-Saharan Africa | 26.45 (19.65-33.27) | 1336.18 (990.11-1681.55) | 47.63 (34.41-62.04) | 1015.82 (728.77-1330.88) |
| Eastern Sub-Saharan Africa | 87.81 (67.84-106.29) | 1211.55 (923.63-1475.79) | 139.69 (109.91-169.67) | 867.14 (676.81-1056.77) |
| Southern Sub-Saharan Africa | 10.5 (6.21-15.61) | 351.94 (207.5-524.21) | 13.23 (7.58-22.52) | 209.82 (118.24-360.89) |
| Western Sub-Saharan Africa | 83.76 (62.32-105.05) | 926.51 (686.93-1165.71) | 127.7 (87.9-170.18) | 676.02 (464.01-900.64) |

HAP-SF, household air pollution from solid fuels; ASMR, age-standardized mortality rate; UI, uncertainty interval.

## **Supplementary Table 4.** HAP-SF-related elderly DALYs and ASDR in 1990 and 2021.

|  | **1990** | | **2021** | |
| --- | --- | --- | --- | --- |
| **Characteristics** | **DALYs** | **ASDR** | **DALYs** | **ASDR** |
|  | **No. ×10^3^ (95% UI)** | **(95% UI)** | **No. ×10^3^ (95% UI)** | **(95% UI)** |
| Overall | 54187.13 (43674.59-65190.1) | 11342.37 (9131.75-13660.14) | 42094.61 (24733.49-72248.54) | 3819.84 (2251.55-6534.18) |
| Sex |  |  |  |  |
| Male | 27969.37 (21874.92-33944.84) | 13416.88 (10477-16310.71) | 21235.38 (12300.8-37918.73) | 4237.87 (2450.52-7581.97) |
| Female | 26217.76 (21063.39-32188.43) | 9746.08 (7832.02-11962.65) | 20859.24 (12370.89-34955.77) | 3465.62 (2071.41-5762.86) |
| Socio-demographic index |  |  |  |  |
| Low | 5835.71 (4797.14-6801.68) | 24214.51 (19795.45-28280.55) | 9901.69 (7999.7-11756.13) | 18508.78 (14906.18-21976.69) |
| Low-middle | 14223.6 (11774.66-16460.04) | 21433.37 (17691.66-24860.73) | 20648.94 (13400.72-28451.57) | 12447.29 (8076.9-17139.75) |
| Middle | 22292.2 (18057.89-26462.2) | 20018.77 (16188.33-23774) | 9890.38 (2555.36-24772.1) | 3036.07 (782.65-7607.39) |
| Middle-high | 10986.11 (7721.86-15239.35) | 9053.64 (6356.11-12576.55) | 1588.99 (156.95-7883.59) | 597.84 (59.18-2962.39) |
| High | 812.49 (275.44-1921.64) | 542.28 (183.82-1283.26) | 37.34 (0.61-270.15) | 12.1 (0.19-88.5) |
| Region |  |  |  |  |
| Central Asia | 470.96 (215.52-863.47) | 8534.13 (3910.49-15630.35) | 227.53 (97.28-499.84) | 2493.74 (1060.25-5500.99) |
| East Asia | 26116.45 (20607.04-31332.89) | 28816.04 (22705.75-34494.68) | 7606.14 (2028.44-22166.47) | 2746.34 (733.12-7998.32) |
| South Asia | 13884 (11428.8-16182.55) | 22775.42 (18683.02-26629.42) | 20824.64 (13627.47-29364.15) | 12080.77 (7894.3-17042.27) |
| Southeast Asia | 5248.94 (4189.82-6267.88) | 19119.93 (15244.69-22850.98) | 4687.07 (2161.06-8165.1) | 6246.92 (2876.94-10864.4) |
| Australasia | 1.15 (0.01-10.61) | 35.96 (0.28-331.81) | 0.14 (-0.01-0.49) | 1.64 (-0.06-6.04) |
| Oceania | 95.15 (73.18-118.99) | 32154.65 (24790.77-40039.2) | 179.8 (133.04-226.22) | 24301.03 (18006.7-30538.83) |
| High-income Asia Pacific | 15.68 (1.33-90.37) | 61.79 (5.22-356.23) | 0.97 (-0.01-4.72) | 1.24 (-0.01-6.05) |
| Central Europe | 904.45 (228.09-2372.76) | 4739.57 (1197.51-12411.69) | 136.52 (7.79-849.4) | 420.36 (23.75-2615.84) |
| Eastern Europe | 417.12 (96.53-1594.2) | 1177.56 (273.04-4487.28) | 127.5 (20.55-518.56) | 258.57 (41.9-1048.02) |
| Western Europe | 30.66 (1.21-217.73) | 37.12 (1.46-264.24) | 2.71 (-0.03-12.56) | 1.77 (-0.02-8.32) |
| Andean Latin America | 141.89 (68.85-226.03) | 5981.64 (2905.3-9522.29) | 72.58 (16.06-206.49) | 972.87 (216.91-2758.07) |
| Central Latin America | 326.93 (136.9-620.09) | 3484.94 (1459.83-6607.37) | 337.13 (121.33-810.51) | 1071.12 (388.11-2563.94) |
| Caribbean | 200.11 (141.54-266.16) | 6237.94 (4412.38-8291.42) | 222.13 (158.57-305.6) | 3313.31 (2369.92-4531.37) |
| High-income North America | 1.96 (0.01-13.11) | 3.94 (0.01-26.5) | 0.67 (-0.03-2.49) | 0.68 (-0.03-2.57) |
| Southern Latin America | 125.81 (33.46-309.93) | 2128.72 (565.88-5245.4) | 10.13 (0.24-82.42) | 83.08 (2.02-678.63) |
| Tropical Latin America | 551.48 (286.93-937.34) | 5404.68 (2819.41-9145.2) | 188.88 (46.8-520.46) | 573.99 (142.57-1583.58) |
| North Africa and Middle East | 1263.61 (815.14-1971.99) | 6911.24 (4427.02-10844.34) | 637.3 (433.58-917.83) | 1281.84 (873.54-1845.35) |
| Central Sub-Saharan Africa | 579.49 (435.31-723.92) | 25512.28 (19186.43-31833.59) | 1006.37 (743.59-1291.86) | 19177.57 (14116.88-24677.39) |
| Eastern Sub-Saharan Africa | 1884.14 (1488.02-2264.01) | 23710.89 (18572.26-28573.28) | 2912.97 (2352.89-3480.21) | 16828.49 (13532.38-20142.16) |
| Southern Sub-Saharan Africa | 218.46 (131.06-318.22) | 7088.67 (4247.95-10335.65) | 282.5 (169.61-471.4) | 4265.04 (2535.09-7156.73) |
| Western Sub-Saharan Africa | 1708.68 (1282.71-2110.27) | 17711.92 (13255.03-21912.36) | 2630.95 (1847.76-3491.1) | 13051.53 (9143.55-17294.52) |

HAP-SF, household air pollution from solid fuels; DALYs, disability-adjusted life years; ASDR, age standardized DALYs rate; UI, uncertainty interval.
